# *Tropheryma whipplei* as the Dominant Organism in Bronchoalveolar-Lavage Metagenomic Next-Generation Sequencing: A Single-Centre Case Series of 28 Patients

**DOI:** 10.3390/diagnostics16193230

**Published:** 2026-10-06

**Authors:** Xi Zhang, Jingdong He, Zhinan Zhai, Xie Zhaoyifan, Xiang Pei, Yi Li, Min Hou

**Affiliations:** 1 Department of Clinical Laboratory, Chest Hospital, Tianjin University, Tianjin 300222, China; 2 Department of Biomedical Sciences, City University of Hong Kong, Hong Kong 999077, China; 3 School of Medical Laboratory, Tianjin Medical University, Tianjin 300070, China; 4 Department of Respiratory and Critical Care Medicine, Chest Hospital, Tianjin University, Tianjin 300222, China

**Keywords:** *Tropheryma whipplei*, dominant organism, read abundance, bronchoalveolar lavage fluid, metagenomic next-generation sequencing, case series, descriptive epidemiology

## Abstract

**Background/Objectives**: Metagenomic next-generation sequencing (mNGS) of bronchoalveolar lavage fluid (BALF) increasingly reports *Tropheryma whipplei* (*T. whipplei*) in patients without classical Whipple disease, but the significance of these detections is often uncertain. We aimed to quantify relative sequencing abundance, characterise the accompanying clinical, radiological, and routine laboratory phenotype, and develop a structured framework for interpretation. **Methods**: We retrospectively reviewed 944 consecutive BALF-mNGS tests performed at Tianjin Chest Hospital between October 2022 and March 2026. *T. whipplei* was detected in 47 tests; the primary analytic cohort comprised the 28 patients in whom it was the most abundant detected microorganism. Demographics, comorbidities, symptoms, chest computed tomography findings, co-detected organisms, species-level read counts, and routine laboratory indicators were summarised descriptively. **Results**: *T. whipplei* was detected in 47/944 tests (4.98%) and was the predominant (most abundant) detected microorganism in 28/47 (59.6%). In these 28 patients, the mean age was 60.2 ± 12.3 years and 15 (53.6%) were women. Cough (18/28, 64.3%) and expectoration (14/28, 50.0%) predominated, whereas fever occurred in 4/28 (14.3%); pulmonary nodules were the most common CT finding (15/28, 53.6%). *T. whipplei* was the sole microorganism in 11/28 (39.3%) and was co-detected in 17/28 (60.7%); it was sole or ≥10-fold more abundant than the strongest co-detected organism in 25/28 (89.3%). Leukocyte counts were within reference in 26/28, C-reactive protein was ≤5 mg/L in 19/25, and procalcitonin was at or below the assay limit in 12/14. **Conclusions**: Predominant (most-abundant) BALF-mNGS detection of *T. whipplei* commonly accompanied nonspecific respiratory presentations and a relatively indolent laboratory phenotype. Because sequence dominance cannot by itself distinguish infection from heavy carriage, interpretation should integrate read burden, orthogonal confirmation, competing pathogens and diagnoses, and systemic features.

## 1. Introduction

*Tropheryma whipplei* (*T. whipplei*) is a reduced-genome actinobacterium that was identified molecularly, from the 16S ribosomal RNA of infected tissue, decades before it could be cultured, and its fastidious growth still makes culturing impractical, so recognition rests on histopathology and molecular methods [1,2,3,4]. The organism is common in the environment and in the human population: asymptomatic carriage is detectable in the stool and saliva of a minority of healthy individuals, yet only a very small fraction of carriers ever develop the disease, a discrepancy attributed to host genetic and immune susceptibility rather than to differences in exposure [2,5,6,7]. Classical Whipple disease—chronic diarrhoea, weight loss, arthralgia and malabsorption—is only one end of a spectrum that also includes asymptomatic carriage, endocarditis, neurological disease and isolated pulmonary involvement [2,8,9,10,11]. Sequencing-based methods have extended detection beyond biopsy to less invasive samples, including plasma microbial cell-free DNA in *T. whipplei* endocarditis [12]. Extra-intestinal presentations are protean and frequently mimic rheumatic disease, which contributes to diagnostic delay [13,14].

Metagenomic next-generation sequencing (mNGS) has changed how the organism is encountered. Because mNGS surveys all nucleic acid in a sample without an a priori hypothesis, it is increasingly used for pathogen detection in bronchoalveolar lavage fluid (BALF), and *T. whipplei* now appears in BALF reports from patients whose clinical question was pneumonia, interstitial disease or a pulmonary nodule [15,16,17,18,19,20]. Reports of respiratory *T. whipplei* detection have accumulated rapidly as the assay has entered routine use, spanning immunocompetent and immunocompromised hosts, coinfections and post-viral settings [21,22,23,24]. The same breadth that makes the assay useful makes its output ambiguous: an analytical detection is not, by itself, evidence of causation. Lin et al. reported the organism in 70 of 1725 BALF samples (4.0%) alongside frequent coexisting diagnoses [17], and in a multicentre series combining mNGS with quantitative polymerase chain reaction (PCR), Sun et al. adjudicated only 14 of 91 respiratory detections (15.4%) as *T. whipplei* pneumonia, classifying the remainder as colonisation, with adjudicated infection tracking higher read burden [25].

Existing reports have concentrated on detection frequency, on the non-specific clinical and imaging picture, and—in the largest series—on adjudicating infection against colonisation or on treatment response [17,25,26,27]. Two elements that would help a clinician read an individual report have been described only patchily: the routine laboratory phenotype—the blood counts, inflammatory markers and biochemistry that constitute almost everything available at the moment the report arrives, alongside a name and a chest computed tomography (CT) scan—and the abundance of the organism relative to everything else in the sample, which is what separates a dominant sequence from a trace detection. Both are precisely the information available when the treat-or-not decision is made, and neither constitutes species-level histology.

Here we report all patients with a positive BALF-mNGS finding of *T. whipplei* at our centre over a 42-month period. We describe who these patients are, how they presented, what else was found in the sample analysed, how abundant the organism was in each report, and, as the primary contribution, their complete routine laboratory profile in the original reported clinical units. Our aim is twofold: to give clinicians an empirical reference picture of this population, and to convert that picture into a structured and more meaningful interpretation.

## 2. Materials and Methods

### 2.1. Design and Case Definition

**Figure 1:**
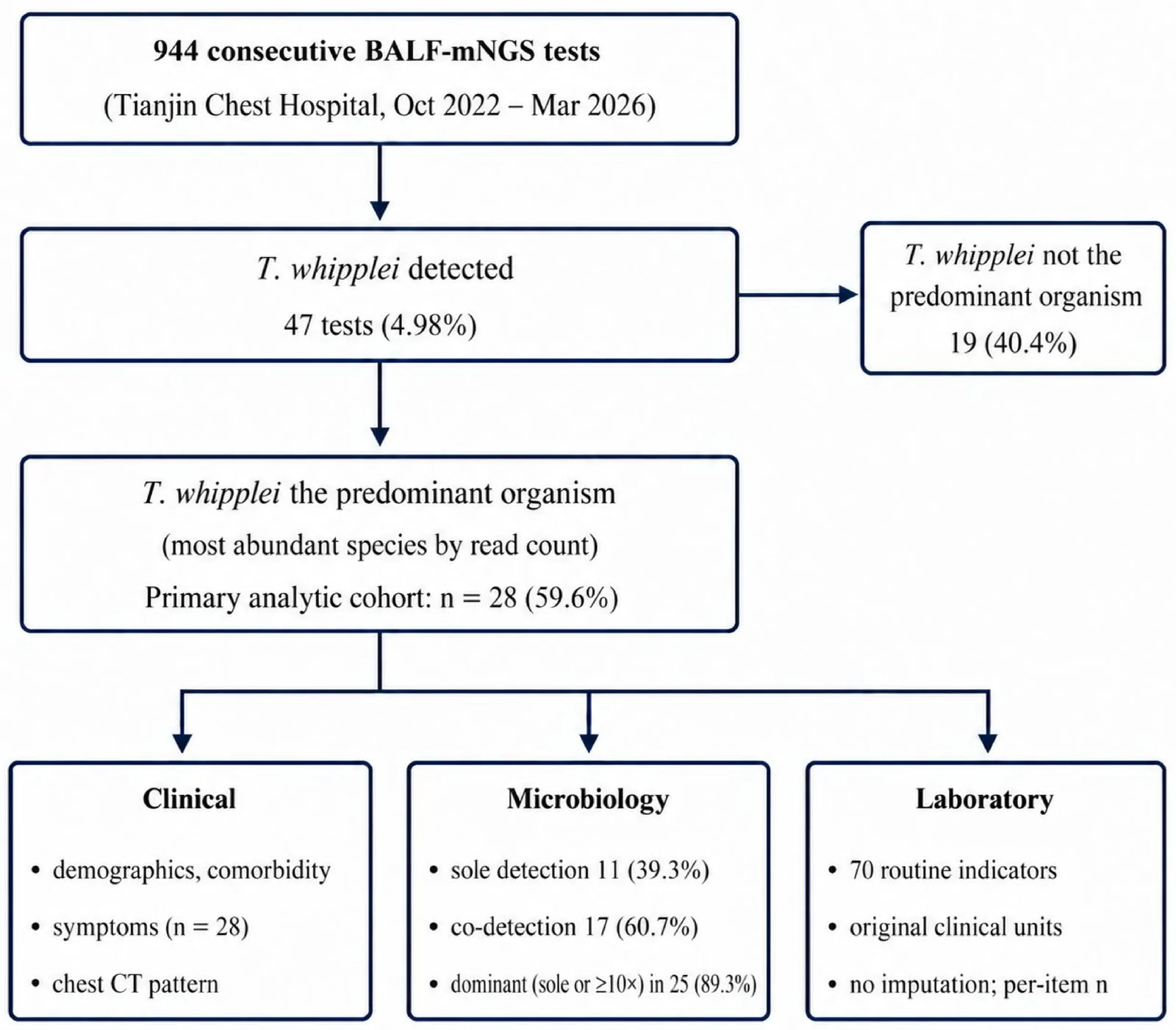
Flow and content of the case series studied. Of 944 consecutive BALFmNGS tests performed at Tianjin Chest Hospital between October 2022 and March 2026, *T. whipplei* was detected in 47 (4.98%); in 28 of these (59.6%) it was the predominant organism—the single most abundant species by read count—and these 28 constitute the case series, whereas in 19 (40.4%) another organism was more abundant. Each patient was characterised along three axes: clinical (demographics, comorbidity, presenting symptoms, chest CT pattern), microbiology (sole detection in 11 [39.3%], co-detection in 17 [60.7%], *T. whipplei* dominant by ≥10×in 25 [89.3%]), and laboratory (70 routine indicators, each summarised on its available denominator in original clinical units, no imputation). No patient contributed more than one detection.

This is a retrospective, consecutive, single-centre case series conducted at Tianjin Chest Hospital and reported in accordance with the PROCESS/CARE principles for case series and the descriptive elements of the STROBE statement. All BALF-mNGS results generated between October 2022 and March 2026 were reviewed (*n* = 944). *T. whipplei* detection was defined by the laboratory as ≥3 non-overlapping species-specific reads in a sample meeting quality thresholds, consistent with previous BALF sequencing studies [16,17]. Using this definition, *T. whipplei* was reported in 47 patients. Among these, the present case series focused on the 28 patients in whom *T. whipplei* was the most abundant detected microorganism; the remaining 19 patients were infected with another organism with a higher read count and were not included in the primary analytic cohort (Figure 1). No patient contributed more than one detection. Thus, in every patient included in the 28-patient series, *T. whipplei* was the predominant organism in the report—the single most abundant species by read count (Section 3.3). Accordingly, the series should be understood not as a collection of threshold-level *T. whipplei* detections, but as a consecutive series of patients in whom *T. whipplei* was the predominant microorganism recovered from BALF. For clarity, we distinguish two terms used throughout this report. *T. whipplei* was designated the predominant (most abundant) organism when it had the highest species-specific read count in the sample; this was the criterion used to select the 28-patient analytic cohort (28/28). The term ‘dominant’ is reserved for the stricter criterion of sole detection or a *T. whipplei* read count at least tenfold higher than that of the strongest co-detected organism, which was met in 25 of the 28 patients (Section 3.3). The denominator of 944 refers to individual BALF-mNGS tests, not unique patients; each of the 47 *T. whipplei*-positive results corresponded to a distinct patient, and no patient contributed more than one positive test during the study period, so for the positive cases tests and patients coincide. These 944 BALF-mNGS tests were performed on 920 unique patients; a minority of patients underwent more than one BALF-mNGS test during the study period, and repeat tests were retained and counted separately in the test-based denominator rather than excluded or restricted to a single episode. All 47 *T. whipplei*-positive results came from 47 distinct patients, with no patient contributing more than one positive test (47/920 unique patients, 5.1%, at the patient level).

### 2.2. BALF Collection and Sequencing

Bronchoalveolar lavage fluid (BALF) was collected by bronchoscopy in accordance with the Chinese expert consensus on pathogen detection in pulmonary infectious dis-eases [28]. Sterile normal saline was instilled into the target bronchopulmonary segment in 20 mL aliquots to a total instilled volume of 100–120 mL; after each aliquot, the fluid was immediately retrieved by negative-pressure suction (25–100 mmHg), a total recovery of more than 30% was ensured, and the recovered fluid was pooled for analysis. Specimens were transported under cold-chain conditions and processed within 24 h. DNA and RNA libraries were prepared separately. Methylated human DNA was depleted from DNA libraries using an antibody-based method, whereas RNA libraries were treated with DNase. Sequencing was performed on an Illumina 550Dx platform (Illumina, Inc., San Diego, CA, USA), targeting 5–20 million reads per library. Quality-control criteria required ≥5 million preprocessed reads per sample and >75% of bases with a quality score of Q30 or higher. All sequencing results underwent laboratory review before release.

The bioinformatic workflow was as follows: raw reads were adapter-trimmed and quality-filtered, and low-quality and short reads were discarded. Human-derived sequences were removed by alignment to the human reference genome (GRCh38) using BWA-MEM2 (version 2.2.1). The remaining non-human reads were taxonomically classified using Kraken (version 2.0.8) against an in-house microbial genome database com-prising bacteria, fungi, viruses, and parasites; the database was last updated on 10 May 2026. Species-level taxonomic assignment required ≥3 non-overlapping species-specific reads. Thus, human-sequence subtraction and low-quality read removal were performed before microbial classification. Detection of *T. whipplei* in this study rested solely on BALF-mNGS; no orthogonal or species-specific confirmatory testing—species-specific quantitative PCR (qPCR), culture, histopathology with periodic acid–Schiff (PAS) staining, or immunohistochemistry—was performed.

### 2.3. Data Extraction and Descriptive Summary

Demographics, comorbidities, presenting symptoms, chest CT findings, co-detected organisms, species-level read counts, treatment and discharge status were extracted from the electronic medical record. Routine laboratory data, including haematological, inflammatory, biochemical, immunological, and coagulation-related parameters, were obtained from the first available test during the same care episode as bronchoscopy and interpreted according to the reference intervals of the reporting laboratory.

Categorical data are reported as counts with the exact denominator available for that item and the corresponding percentage. Continuous laboratory data are reported as median (IQR) and full range in original clinical units, with the number of values above and below the sex-specific reference interval of the reporting laboratory. Read burden was summarised for each patient as the *T. whipplei* read count, its share of the total microbial reads in the report, and—where any other organism was reported—the ratio of *T. whipplei* reads to the reads of the most abundant co-detected organism. A detection was labelled *dominant* when *T. whipplei* was the only organism or its reads were ≥10×those of the strongest co-pathogen. No transformation was applied to any reported value. Absolute read burden (the *T. whipplei* read count) and relative dominance (the ratio of *T. whipplei* reads to those of the strongest co-detected organism) were evaluated separately, because a sole detection based on very few reads is biologically distinct from a high-burden detection even when both satisfy a dominance rule. The tenfold ratio was chosen a priori as a conservative, order-of-magnitude margin for identifying the principal organism in a sample; because any single cut-off is arbitrary, we additionally report the continuous read-ratio distribution and a threshold sensitivity analysis (Section 3.3) rather than relying on the tenfold value alone.

Values reported by the laboratory below an assay limit (for example “<0.05 ng/mL” for procalcitonin) were reported as being at the limit of detection.

## 3. Results

### 3.1. Frequency and Cohort Composition

Structural or chronic airway/lung disease was uncommon in this cohort: no patient had a documented diagnosis of chronic obstructive pulmonary disease or bronchiectasis, and lung cancer was present in 4 of 28 patients (14.3%); the remainder presented predominantly with pulmonary opacities/pneumonia rather than chronic structural airway disease. The near-absence of chronic airway disease makes colonisation of structurally damaged airways an unlikely blanket explanation for these detections, although it does not by itself distinguish infection from colonisation—particularly in the four patients with lung cancer, in whom post-obstructive change and local immune compromise warrant added caution.

*T. whipplei* was detected in 47 of 944 consecutive BALF-mNGS tests (4.98%; Figure 1). Among these 47 cases, *T. whipplei* was the predominant (most abundant) detected microorganism in 28 (59.6%), who constituted the primary analytic cohort. In these 28 patients, the mean age was 60.2 ± 12.3 years (median 64, IQR 52–69, range 37–79), and 15 (53.6%) were women. Four patients (14.3%) had a smoking history and five (17.9%) reported alcohol use. Hypertension was present in 14 patients (50.0%), diabetes mellitus in 8 (28.6%), and rheumatoid arthritis in 1 (3.6%) (Table 1).

### 3.2. Presentation and Imaging

One patient was identified incidentally and was asymptomatic. Cough was reported by 18 patients (64.3%) and expectoration by 14 (50.0%), followed by dyspnoea or wheezing in 7 (25.0%), chest tightness in 4 (14.3%), fever in 4 (14.3%) and haemoptysis or blood-streaked sputum in 2 (7.1%). Chronic diarrhoea, weight loss, arthralgia and malabsorption—the classical Whipple-disease syndrome—were not present in any patient.

**Figure 2:**
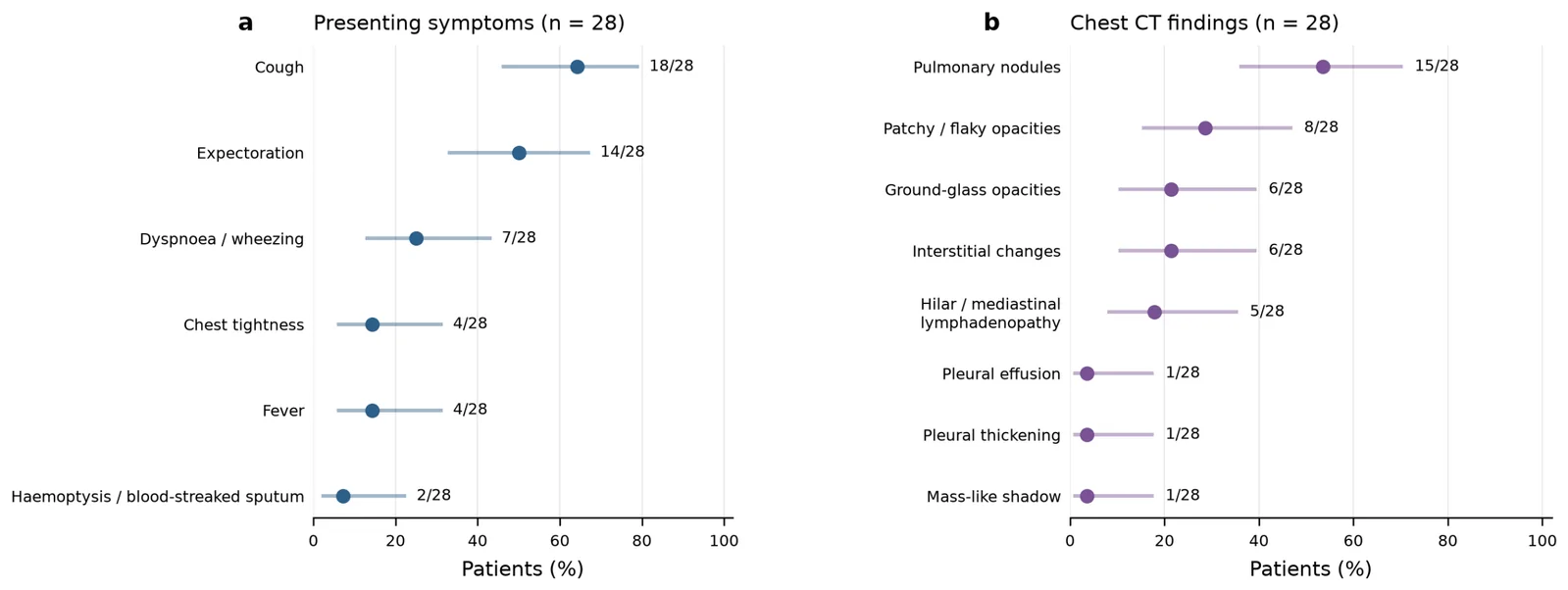
Descriptive frequencies in the 28-patient series with Wilson 95% confidence intervals. (**a**) Presenting symptoms. (**b**) Chest CT findings. Intervals are wide throughout; features observed in one or two patients are compatible with frequencies from under 1% to nearly 20%.

Chest CT showed pulmonary nodules in 15 patients (53.6%), patchy or flaky opacities in 8 (28.6%), ground-glass opacities in 6 (21.4%), interstitial changes in 6 (21.4%) and hilar or mediastinal lymphadenopathy in 5 (17.9%); pleural effusion, pleural thickening and a mass-like shadow occurred in one patient each. Nodules were thus the only finding present in more than half the series, and the confidence interval around every other pattern includes values below 40% (Figure 2a,b and Table 1).

### 3.3. Co-Detected Organisms and Read-Level Dominance

*T. whipplei* was the only organism named in 11 patients (39.3%). The remaining 17 (60.7%) had at least one additional detection, but no single co-pathogen predominated: the most abundant co-detected organism was *Staphylococcus aureus*, *Haemophilus influenzae*, *Mycoplasma pneumoniae* or *Candida albicans* in two patients each and a different organism—bacterial, fungal or viral—in each of the remaining nine (Table 2).

**Figure 3:**
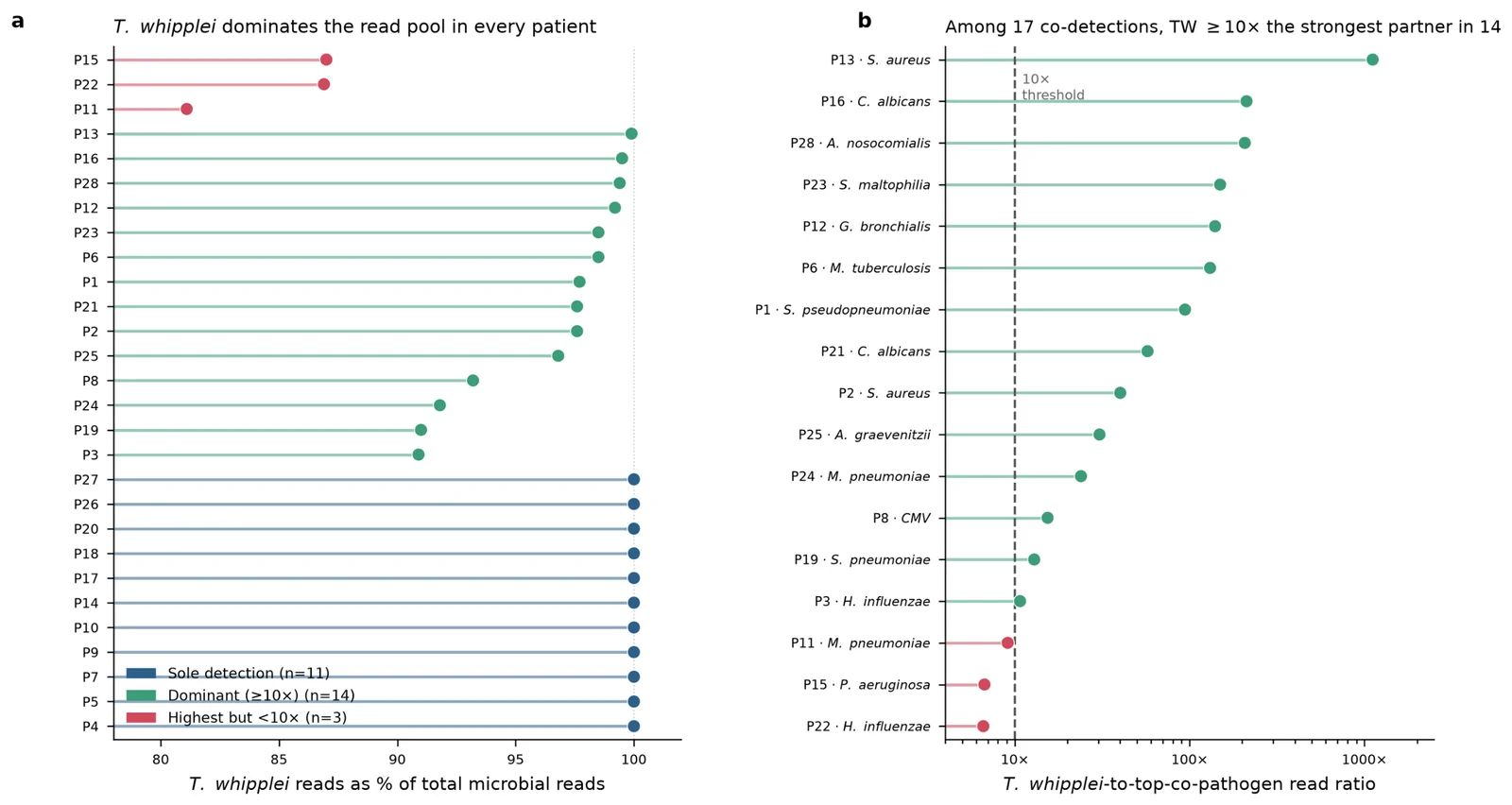
Read-level dominance of *T. whipplei* in the 28 reports. (**a**) *T. whipplei* as a percentage of total microbial reads in each patient, coloured by dominance status (sole detection; dominant, ≥10× the strongest co-pathogen; or highest but <10×). (**b**) Ratio of *T. whipplei* reads to those of the most abundant co-detected organism among the 17 patients with a co-detection, on a logarithmic axis, with the tenfold dominance threshold marked. *T. whipplei* was the most abundant organism in all 28 patients and dominant in 25 (89.3%). Green indicates a *T. whipplei*-to-co-pathogen read ratio ≥10×, whereas red indicates a ratio <10×. The dashed line represents the 10× threshold.

Read counts placed these detections on a scale of relative abundance and gave the series its most consistent microbiological feature. *T. whipplei* was the single most abundant organism in every patient (28/28, 100%), accounting for a median 99.3% of the microbial reads in the report (IQR 95.9–100.0, range 81.1–100.0). The *T. whipplei* read count itself spanned four orders of magnitude (median 1522, IQR 312–17,159, range 3–209,631), so the detections ranged from a handful of reads to a near-clonal signal. Among the 17 patients with a co-detection, *T. whipplei* reads exceeded those of the strongest co-pathogen by a median factor of 40 (IQR 12.9–139.8, range 6.6–1115.1) and by ≥10×in 14 of them (82.4%). Combining sole detections with these dominant co-detections, *T. whipplei* was dominant—the sole or ≥10×the nearest organism—in 25 of 28 patients (89.3%); in only three patients (10.7%) was it the most abundant organism by a margin under tenfold (Figure 3 and Table 2). A detection that is the dominant sequence in the sample in nine of ten patients is not readily dismissed as incidental low-level carriage, whatever its ultimate clinical significance. To assess sensitivity to the dominance threshold, we varied the read-ratio cut-off. Because the smallest *T. whipplei*-to-co-pathogen ratio among the 17 co-detected patients was 6.6, all 28 patients met a ≥5 X criterion, 25 met the ≥10×criterion applied here, and progressively fewer met higher cut-offs (median ratio 40; IQR 12.9–139.8; range 6.6–1115.1). Treated as a continuous measure, *T. whipplei* accounted for a median 99.3% of microbial reads (IQR 95.9–100.0). The qualitative conclusion—that *T. whipplei* was usually the principal microbial constituent of the lavage—was therefore robust across thresholds, whereas the exact number labelled dominant depends on the chosen cut-off. We emphasise that this read-level dominance was not corroborated by any orthogonal method (no species-specific qPCR, culture, histopathology/PAS staining, or immunohistochemistry was performed); the read-based categories describe relative sequencing abundance only and cannot by themselves establish invasive infection.

### 3.4. Routine Laboratory Profile

A total of 70 routine laboratory indicators were extracted, of which 68 had at least one available value. Data availability varied substantially across indicators: full blood count and liver and renal biochemistry were available in 27–28 patients, whereas glycated haemoglobin was available in only 7 patients and the lipid panel in 13–14. Table 3 provides the complete descriptive summary of the 68 indicators with available data, reported in original clinical units together with the reference interval, available denominator, median (IQR), full range, and the number of values outside the reference range.

**Figure 4:**
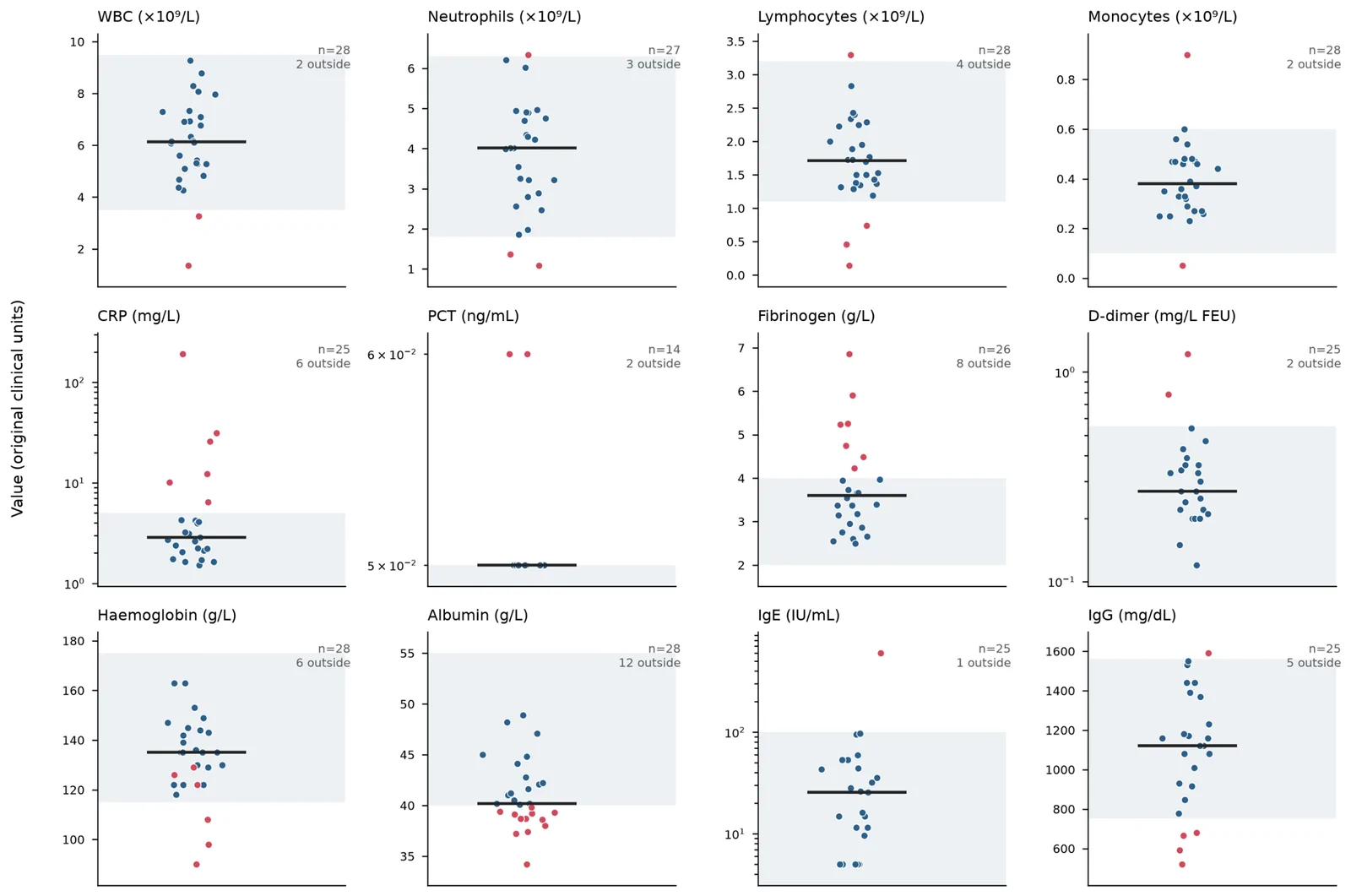
Individual values for twelve core laboratory indicators. Each point represents one patient; the shaded band is the sex-specific reference interval of the reporting laboratory; blue points lie within the reference interval, whereas red points lie outside it; the horizontal bar is the median. Denominators differ between panels and are printed in each. Logarithmic axes are used for CRP, procalcitonin, IgE and D-dimer. No between-group comparison is made.

Markers of systemic inflammation were mostly unremarkable. The leukocyte count was within the reference range in 26 of 28 patients (median 6.14 × 10^9^/L, IQR 5.23–7.14), with no patient above the upper limit. C-reactive protein was ≤5 mg/L in 19 of 25 patients with an available result (median 2.85 mg/L, IQR 2.11–4.25); the six elevated values ranged from 6.44 to 192 mg/L, with the highest value occurring in a patient with a co-detected bacterial pathogen. Procalcitonin was at or below the assay reporting limit of 0.05 ng/mL in 12 of 14 patients, and the remaining two values were both 0.06 ng/mL; this indicator was therefore minimally informative in this series, and its median should not be interpreted as a meaningful central estimate. Neutrophil count was within the reference range in 24 of 27 patients and monocyte count in 26 of 28 (Figure 4).

Coagulation indices showed one relatively frequent abnormality. Fibrinogen was above 4 g/L in 8 of 26 patients (30.8%; median 3.61 g/L, IQR 3.00–4.43, range 2.50–6.86), whereas D-dimer was above the reference range in only 2 of 25 patients (median 0.27 mg/L FEU, IQR 0.22–0.36). Prothrombin time, INR, and activated partial thromboplastin time were within the reference range in the large majority of patients.

The most frequent abnormalities were metabolic and nutritional rather than inflammatory. Albumin was below 40 g/L in 12 of 28 patients (42.9%; median 40.2 g/L, IQR 39.0–42.4), and total protein was below 65 g/L in 12 of 27 (44.4%), while globulin was below the reference range in 5 of 28. Glucose exceeded the upper reference limit in 10 of 25 patients (40.0%; median 5.56 mmol/L, range 3.22–16.15), consistent with the 28.6% prevalence of diabetes mellitus. Glycated haemoglobin, available in only seven patients, was above 6% in six of them; because of the limited and selected availability of this test, these values should not be considered representative of the whole series. Estimated glomerular filtration rate was below 90 mL/min/1.73 m^2^ in 9 of 27 patients. These laboratory patterns are descriptive and are reported without a comparison group; in the absence of a *T. whipplei*-negative comparator they cannot be attributed specifically to *T. whipplei* and may in part reflect the population selected for bronchoscopy and mNGS.

Red-cell and immunoglobulin indices also showed limited and heterogeneous abnormalities. Haemoglobin was below the sex-specific reference limit in 6 of 28 patients (median 135 g/L, IQR 122–143, range 90–163), and haematocrit was below the reference in 7 of 28; mean corpuscular volume was within the reference range in 26 of 27 patients, indicating that the observed anaemia was predominantly normocytic. Immunoglobulin G was below the reference range in 4 of 25 patients and IgM in 4 of 25; IgE exceeded 100 IU/mL in only 1 of 25 patients (median 25.7 IU/mL, IQR 11.5–44.0). Rheumatoid factor was below the limit of quantification in most patients, with two elevated values (91.6 and 568 IU/mL), one of which occurred in the patient with known rheumatoid arthritis.

### 3.5. Between-Patient Heterogeneity

**Figure 5:**
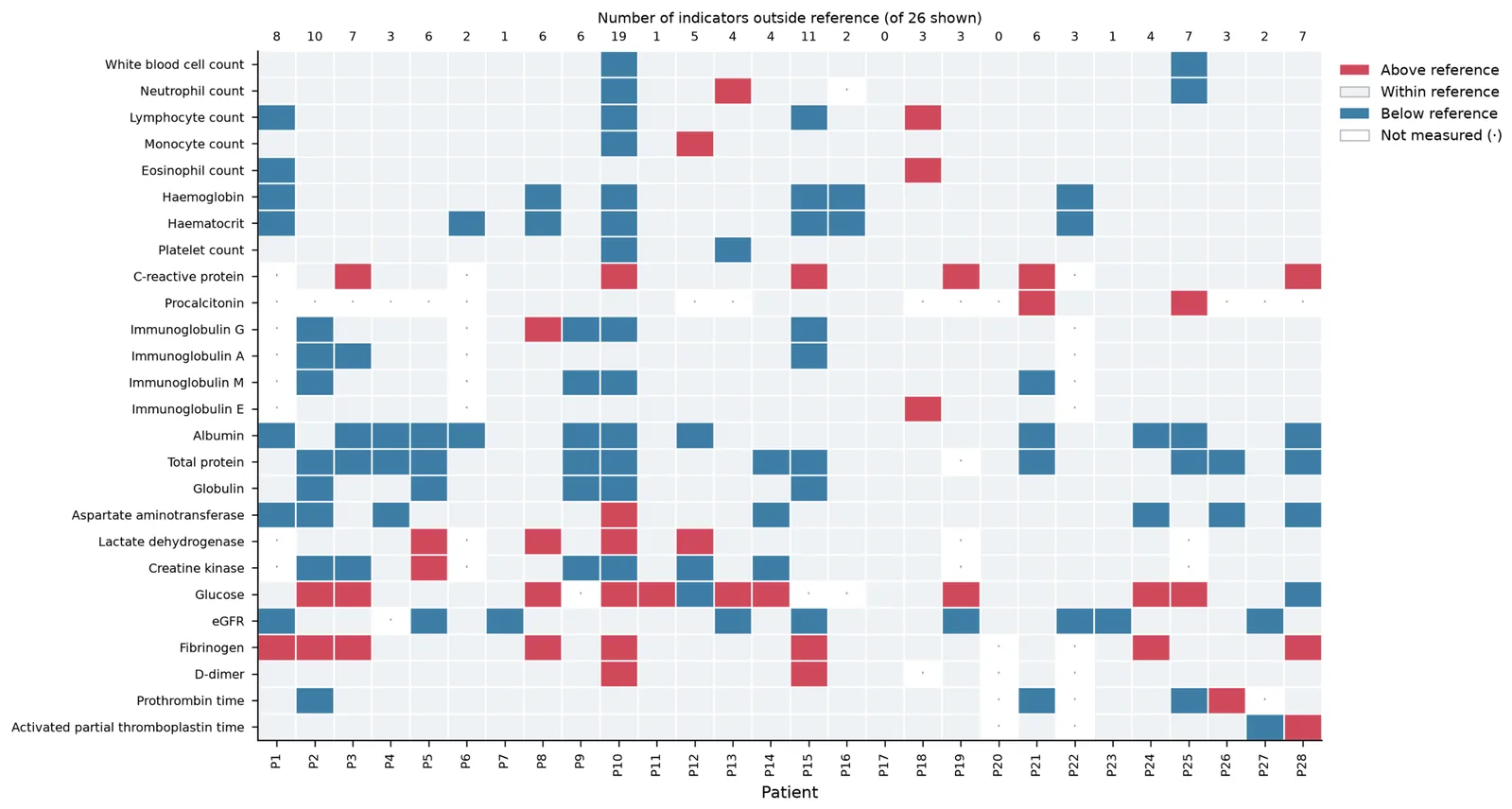
Per-patient abnormality matrix for 26 core indicators. Each column is one patient and each row one indicator; cells are coloured by position relative to the sex-specific reference interval, with a dot marking values not measured. The top axis gives the number of indicators outside the reference for each patient (median 4, IQR 2–6, range 0–19).

Aggregate medians conceal wide individual variation. Across 26 core indicators available in most patients, the median patient had 4 values outside the reference range (IQR 2–6, full range 0–19). Two patients had no abnormality at all among these indicators, eight had two or fewer, and three had ten or more. The single patient with 19 abnormal values had a sole *T. whipplei* detection, whereas the highest C-reactive protein in the series occurred in a different patient who carried a co-detected bacterial pathogen—a reminder that neither the breadth of laboratory disturbance nor the inflammatory response mapped simply onto the microbiological picture. Figure 5 displays every patient and indicator individually, so that the reader can see which combinations occurred together rather than only their marginal frequencies. No recurring cluster of abnormalities was apparent across patients, and with 28 patients we make no attempt to define phenotypic subgroups.

### 3.6. Treatment and Outcome

Management was heterogeneous and reflected the clinical uncertainty that a *T. whipplei* report creates. Some patients received no *T. whipplei*-directed therapy because an alternative diagnosis was considered more plausible; others improved on empirical antibacterial or antifungal therapy directed at a co-detected or clinically dominant pathogen. Because treatment allocation was not protocolised and follow-up was not standardised, we describe these patterns without inferring treatment effect, and we do not report response rates.

## 4. Discussion

This consecutive series describes what a BALF-mNGS report of *T. whipplei* looked like in practice at one Chinese chest hospital over 42 months: what the routine laboratory phenotype of these patients actually is, how abundant the organism was within each report, and how the two might be interpreted together.

Our *T. whipplei* detection frequency of 4.98% (47/944) was comparable to the 4.0% reported by Lin et al. [17] and the 3.98% reported by Jiang et al. [29]. The similarity across independent Chinese cohorts suggests that BALF *T. whipplei* detection is a reproducible finding in respiratory mNGS rather than a local artefact. Longer-running single-centre PCR cohorts have likewise accumulated cases only gradually, indicating that positive detections remain relatively uncommon in absolute terms [30]. Reports of *T. whipplei* in BALF from geographically distinct populations, including a recent series from the Americas, further suggest that its detection is not geographically restricted and may increasingly reflect the broader use of untargeted sequencing rather than a true increase in incidence [31]. At a frequency of approximately one in twenty BALF-mNGS tests, *T. whipplei* is likely to be encountered periodically in a busy bronchoscopy service, underscoring the practical value of a consistent framework for interpreting such detections.

Neither the clinical nor the radiological picture identified these patients in advance. Cough and pulmonary nodules predominated, but both are among the most common reasons for bronchoscopy in the first place, and the classical multisystem Whipple syndrome was absent throughout. This matches the published respiratory experience [15,16,17,18,19], and it means that *T. whipplei* will continue to be found chiefly by untargeted sequencing rather than by clinical suspicion. Contemporaneous BALF-mNGS cohorts describe the same picture: in a case–control comparison Shen et al. found the clinical and imaging features of *T. whipplei*-detected patients to be largely non-specific [26], and the retrospective case series of Yang et al. likewise reported cough and non-specific infiltrates rather than a recognisable syndrome [27]. These comparisons are descriptive; the cohorts differ in setting and case definition, and we do not attempt a formal between-study test. The practical consequence is that the interpretive work has to be done after the report arrives, because nothing can be done before it flags these patients.

The routine laboratory picture in most of these patients was not that of an acute pyogenic pneumonia, and making this phenotype explicit is the most immediately useful offer of the series. Leukocyte counts, CRP and procalcitonin were within or near the reference in the majority, while the recurring abnormalities were hypoalbuminaemia, hypoproteinaemia and hyperglycaemia—a metabolic–nutritional rather than an inflammatory signature. A clinician who sees a normal CRP and procalcitonin in a *T. whipplei*-positive patient now has an empirical reference showing that this is the rule, not a reason to dismiss the finding. Two readings of the pattern are compatible with the data. One is biological: *T. whipplei* has an intracellular lifestyle and has been linked to altered macrophage and T-helper-cell responses [5,6,7,8], so a muted peripheral inflammatory response would be unsurprising. The other is selection: mNGS is often ordered precisely when conventional workup has been unrevealing and the patient is not floridly septic, so an indolent laboratory profile may partly reflect who gets tested. Our data cannot separate these, and a case series is not the design that could; but either way the phenotype is what the clinician will meet, and it argues against equating a *T. whipplei* detection with bacterial pneumonia by reflex. Accordingly, we describe this metabolic–nutritional pattern as an observation in this selected series rather than as a signature attributable to *T. whipplei*; because there was no comparator group, we cannot determine whether it is specific to *T. whipplei* detection or is common to patients undergoing BALF-mNGS for unresolved respiratory illness.

Whether the detection represents infection or colonisation is the question the report cannot answer by itself, and two features of our data pull in opposite directions. Co-detection was frequent—60.7% of patients carried at least one additional organism—so a *T. whipplei* line is often not the whole microbiological story. Yet read counts show that *T. whipplei* was nonetheless the dominant sequence in the sample in almost every case: the most abundant organism in all 28 patients and ≥10× the nearest competitor in 25. Sun et al., combining mNGS with qPCR across multiple centres, adjudicated only 15.4% of respiratory detections as pneumonia and attributed the remainder to colonisation, with pneumonia cases carrying higher read counts [25]; sequential mNGS during treatment has likewise shown burden falling by orders of magnitude as patients respond [27]. Read burden is thus the single most discriminating variable available, and our finding that it is high and dominant in most patients argues against reading the typical report as incidental low-level carriage—even though dominance alone cannot prove causation, since a heavily colonising organism can also dominate a sample. What dominance does establish is that the assay is usually reporting the principal microbial constituent of the lavage, not a trace signal at the edge of detection; the three patients in whom *T. whipplei* led by less than tenfold are precisely those in whom a competing pathogen deserves the closer look. Orthogonal or quantitative approaches—species-specific qPCR, targeted or hybrid-capture sequencing—remain the way to convert this abundance signal into a specific diagnosis [23,32,33]. Luo et al.’s report of an immunocompetent woman with mNGS-diagnosed *T. whipplei* pneumonia who recovered on moxifloxacin monotherapy [34] shows that genuine pulmonary infection does occur; the clinical problem is telling it apart from heavy carriage in the individual patient. We reiterate that no orthogonal confirmation (species-specific qPCR, culture, histopathology with PAS staining, or immunohistochemistry) was available in this series; a positive read-level signal—however dominant—therefore supports the presence of the organism but does not establish invasive pulmonary infection.

Treatment of confirmed disease remains largely extrapolated from experience with classical Whipple disease, where prolonged antibiotic therapy is standard; a recent randomised trial reported that oral doxycycline plus hydroxychloroquine was non-inferior to intravenous ceftriaxone followed by oral co-trimoxazole, supporting an all-oral regimen for systemic disease [35]. Whether isolated pulmonary detection warrants the same duration and combination is unresolved: reported respiratory cases have variously received fluoroquinolone monotherapy, co-trimoxazole or doxycycline-based regimens with apparent recovery [23,27,34], and no pulmonary-specific treatment standard exists. In one series, failure of empirical broad-spectrum therapy followed by rapid defervescence targeted anti-*T. whipplei* treatment was added and used as a bedside argument for genuine infection [27]. Our series was not designed to evaluate therapy and we did not attempt to relate regimen to outcome, so we draw no treatment conclusions from our own data.

**Figure 6:**
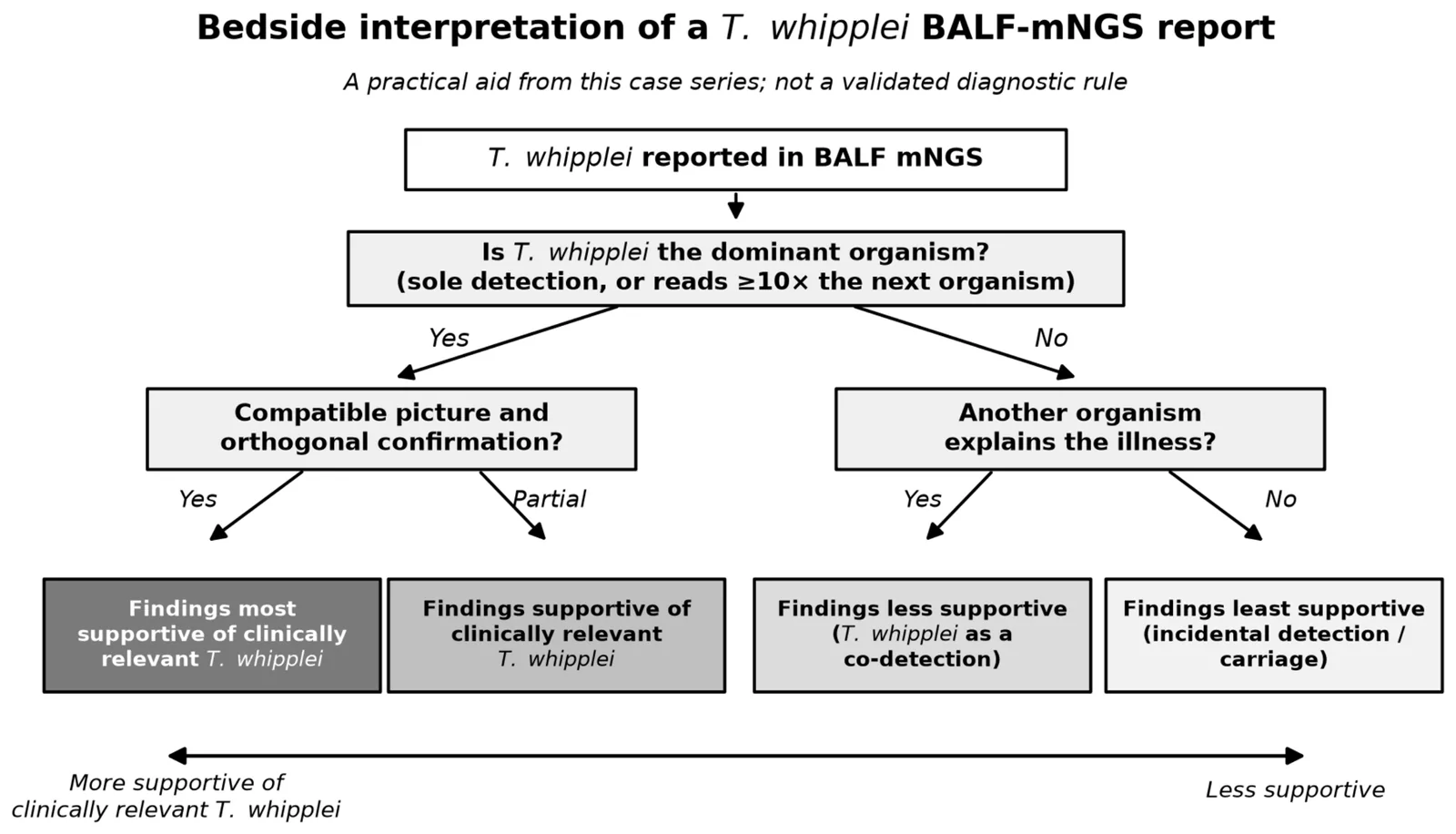
Bedside interpretation of a *T. whipplei* BALF-mNGS report, as a decision tree. Two sequential questions—whether *T. whipplei* is the dominant organism (sole detection or reads ≥10× the next organism), and, on each branch, whether the clinical picture and orthogonal confirmation support it or whether another organism explains the illness—sort the report along a gradient from findings more supportive to less supportive of clinically relevant *T. whipplei,* resolving to one of four interpretive levels (findings most supportive; findings supportive; findings less supportive, *T. whipplei* as a co-detection; or findings least supportive, incidental detection/carriage). It is offered as a practical aid for consistent interpretation, assembled from the present series and the published experience, not as a validated diagnostic algorithm. A dominant read-level signal or a positive quantitative assay supports the presence of the organism but does not by itself establish invasive infection; the categories represent an interpretive gradient only and do not constitute diagnostic verdicts or treatment recommendations. The grey shading from dark to light represents decreasing support for clinically relevant *T. whipplei*.

Taken together, these observations suggest that a positive mNGS finding of *T. whipplei* should trigger a structured assessment rather than an automatic, unplanned treatment regimen and/or dismissal. The elements are read burden and dominance, which our series shows are usually high and which place the detection on a scale rather than treating it as a yes/no result; with orthogonal confirmation by species-specific qPCR; an explicit judgement about competing pathogens and competing diagnoses; the laboratory phenotype described herein; and—where systemic features are present—consideration of duodenal biopsy with periodic acid–Schiff staining. Figure 6 sets these out as a practical pathway that resolves to one of four interpretive levels, ordered from findings most supportive to least supportive of clinically relevant *T. whipplei:* findings most supportive, findings supportive, findings less supportive (*T. whipplei* as a co-detection), and findings least supportive (incidental detection/carriage). We present this as a descriptive aid to consistent interpretation, assembled from our observations and the published experience, not as a validated diagnostic rule; making assessments comparable between centres is its purpose, and validating it would require the prospective data set out below. The four interpretive levels in Figure 6 are descriptive categories intended to structure clinical reasoning, not diagnostic verdicts; the most supportive category in particular would require orthogonal confirmation and exclusion of competing causes; the figure presents an interpretive gradient only and does not carry treatment recommendations.

## 5. Limitations

Several limitations bound what this series can claim, and they also define the axes of the assessment pathway in Figure 6. The study is retrospective, single-centre and small, describing only patients tested at a specialist chest hospital, so both the frequency and the clinical mix are specific to that setting. There is no comparison group by design; nothing here establishes that any feature is more or less common in *T. whipplei*-positive than in *T. whipplei*-negative patients, and we deliberately undertook no between-group test or predictive model. Detection rested on the reporting laboratory’s threshold without orthogonal confirmation: no species-specific qPCR, duodenal biopsy, periodic acid–Schiff staining, immunohistochemistry or culture was performed, so although read counts show that *T. whipplei* was the most abundant organism in almost every sample, read-level dominance cannot by itself distinguish infection from heavy colonisation in an individual case. Treatment was not protocolised and structured follow-up (relapse, microbiological clearance, radiological resolution) was unavailable, so outcomes are described only narratively. In particular, no species-specific qPCR, culture, histopathology with periodic acid–Schiff staining, or immunohistochemistry was performed (as also stated in the Methods and Results); accordingly, no finding in this series should be over-interpreted as confirming invasive *T. whipplei* infection.

What would answer the questions this series raises is a prospective multicentre study with a standardised respiratory case definition, quantitative *T. whipplei* PCR on every positive sample, full read-count reporting, prospectively matched comparators, and longitudinal follow-up.

## 6. Conclusions

Among 944 consecutive BALF-mNGS tests, *T. whipplei* was detected in 47 cases (4.98%), and was the predominant (most abundant) detected microorganism in 28 of these cases (59.6%). The immediate value of this series lies in providing a practical reference profile for clinicians interpreting a BALF-mNGS report containing *T. whipplei*. In the 28 patients with predominant *T. whipplei* detection, clinical presentation was characterised by nonspecific respiratory symptoms and predominantly nodular CT findings, with frequent microbial co-detections. Routine laboratory findings more often reflected an indolent metabolic and nutritional disturbance than an acute systemic inflammatory response, with hypoalbuminaemia and hyperglycaemia among the most frequent abnormalities and substantial between-patient heterogeneity, ranging from no abnormal indicators to nineteen. These laboratory observations are descriptive and uncontrolled and should not be read as a *T. whipplei*-specific signature.

The finding that *T. whipplei* represented the most abundant microorganism in nearly 60% of all positive detections suggests that at least a substantial proportion of BALF-mNGS signals cannot be readily dismissed as low-level incidental findings. Nevertheless, sequence dominance alone does not distinguish active pulmonary infection from substantial airway carriage. Accordingly, detection of *T. whipplei* should not be interpreted as a diagnosis in isolation, but rather as one component of a structured assessment incorporating sequencing burden and relative dominance, orthogonal microbiological confirmation, competing pathogens and alternative diagnoses, the routine laboratory phenotype, and evidence of systemic involvement, ultimately supporting assignment to one of four interpretive levels (Figure 6).

This descriptive framework provides a reproducible approach for interpreting individual *T. whipplei*-positive BALF-mNGS reports. Prospective studies incorporating quantitative confirmation, standardised clinical adjudication, and adequate sample sizes are required to determine which detections represent clinically relevant infection and to validate the proposed interpretive framework.

## Figures and Tables

**Table 1:** Demographic, clinical, and radiological characteristics of the 28-patient primary analytic cohort.

Category	Characteristic	*n* (%) or Value	95% CI (%)
Demographics	Age, years—mean ± SD	60.2 ± 12.3	
Demographics	Female sex	15 (53.6)	35.8–70.5
Demographics	Male sex	13 (46.4)	29.5–64.2
Comorbidity	Hypertension	14 (50.0)	32.6–67.4
Comorbidity	Diabetes mellitus	8 (28.6)	15.3–47.1
Comorbidity	Rheumatoid arthritis	1 (3.6)	0.6–17.7
History	Smoking history	4 (14.3)	5.7–31.5
History	Alcohol history	5 (17.9)	7.9–35.6
Symptoms	Cough	18 (64.3)	45.8–79.3
Symptoms	Expectoration	14 (50.0)	32.6–67.4
Symptoms	Dyspnoea/wheezing	7 (25.0)	12.7–43.4
Symptoms	Chest tightness	4 (14.3)	5.7–31.5
Symptoms	Fever	4 (14.3)	5.7–31.5
Symptoms	Haemoptysis/blood-streaked sputum	2 (7.1)	2.0–22.6
Symptoms	Asymptomatic	1 (3.6)	0.6–17.7
Symptoms	Classical Whipple syndrome	0 (0.0)	0.0–12.1
Chest CT	Pulmonary nodules	15 (53.6)	35.8–70.5
Chest CT	Patchy/flaky opacities	8 (28.6)	15.3–47.1
Chest CT	Ground-glass opacities	6 (21.4)	10.2–39.5
Chest CT	Interstitial changes	6 (21.4)	10.2–39.5
Chest CT	Hilar/mediastinal lymphadenopathy	5 (17.9)	7.9–35.6
Chest CT	Pleural effusion	1 (3.6)	0.6–17.7
Chest CT	Pleural thickening	1 (3.6)	0.6–17.7
Chest CT	Mass-like shadow	1 (3.6)	0.6–17.7

**Table 2:** mNGS detection pattern and read-level dominance: sole detection versus co-detection, dominance status, and the most abundant co-detected organism in the co-detected subgroup.

Group	Item	*n* (%)	95% CI (%)
Detection pattern	*T. whipplei* sole detection (no co-detected organism)	11 (39.3)	23.6–57.6
Detection pattern	≥1 co-detected organism	17 (60.7)	42.4–76.4
Dominance status	Sole detection	11 (39.3)	23.6–57.6
Dominance status	Dominant (≥10×)	14 (50.0)	32.6–67.4
Dominance status	Highest but <10×	3 (10.7)	3.7–27.2
Dominance status	*T. whipplei* = highest-abundance organism (any)	28 (100.0)	87.9–100.0
Dominance status	Dominant overall (sole or ≥10×)	25 (89.3)	72.8–96.3
Most abundant co-pathogen (co-detected subgroup, *n* = 17)	*S. aureus*	2 (11.8)	3.3–34.3
Most abundant co-pathogen (co-detected subgroup, *n* = 17)	*H. influenzae*	2 (11.8)	3.3–34.3
Most abundant co-pathogen (co-detected subgroup, *n* = 17)	*M. pneumoniae*	2 (11.8)	3.3–34.3
Most abundant co-pathogen (co-detected subgroup, *n* = 17)	*C. albicans*	2 (11.8)	3.3–34.3
Most abundant co-pathogen (co-detected subgroup, *n* = 17)	*S. pseudopneumoniae*	1 (5.9)	1.0–27.0
Most abundant co-pathogen (co-detected subgroup, *n* = 17)	HHV-5 (CMV)	1 (5.9)	1.0–27.0
Most abundant co-pathogen (co-detected subgroup, *n* = 17)	*M. tuberculosis*	1 (5.9)	1.0–27.0
Most abundant co-pathogen (co-detected subgroup, *n* = 17)	*G. bronchialis*	1 (5.9)	1.0–27.0
Most abundant co-pathogen (co-detected subgroup, *n* = 17)	*P. aeruginosa*	1 (5.9)	1.0–27.0
Most abundant co-pathogen (co-detected subgroup, *n* = 17)	*S. pneumoniae*	1 (5.9)	1.0–27.0
Most abundant co-pathogen (co-detected subgroup, *n* = 17)	*S. maltophilia*	1 (5.9)	1.0–27.0
Most abundant co-pathogen (co-detected subgroup, *n* = 17)	*A. graevenitzii*	1 (5.9)	1.0–27.0
Most abundant co-pathogen (co-detected subgroup, *n* = 17)	*A. nosocomialis*	1 (5.9)	1.0–27.0

**Table 3:** Complete summary of routine laboratory indicators in original reported clinical units—reference interval, number of patients with an available result, median (IQR), full range, values above and below reference, and number of left-censored values.

Category	Indicator (Unit)	*n* Available/28	Reference Interval	Median (IQR)	Range	Above Reference, *n* (%)	Below Reference, *n* (%)	Left-Censored Values, *n*
Full blood count	Basophil count (×10^9^/L)	28	0–0.06	0.035 (0.02–0.04)	0.01–0.06	0 (0.0)	0 (0.0)	
Full blood count	Eosinophil count (×10^9^/L)	28	0.02–0.52	0.13 (0.08–0.1925)	0.01–0.73	1 (3.6)	1 (3.6)	
Full blood count	Haematocrit (%)	28	M 40–50; F 35–45	39.7 (37.7–43.15)	28.1–49.6	0 (0.0)	7 (25.0)	
Full blood count	Haemoglobin (g/L)	28	M 130–175; F 115–150	135 (122–143.25)	90–163	0 (0.0)	6 (21.4)	
Full blood count	Immature granulocyte count (×10^9^/L)	28	—	0.01 (0.01–0.02)	0–0.07	—	—	
Full blood count	Large platelet ratio (%)	28	—	23.25 (16.925–27.8)	13.5–41.3	—	—	
Full blood count	Lymphocyte count (×10^9^/L)	28	1.1–3.2	1.715 (1.365–2.235)	0.14–3.3	1 (3.6)	3 (10.7)	
Full blood count	MCHC (g/L)	28	—	332 (325.25–338.25)	314–343	—	—	
Full blood count	Mature neutrophil count (×10^9^/L)	24	—	3.975 (2.855–4.755)	1.07–6.27	—	—	
Full blood count	Mean corpuscular haemoglobin (pg)	27	—	30.2 (29.45–31.05)	27.5–33.6	—	—	
Full blood count	Mean corpuscular volume (fL)	27	82–100	91.7 (89.45–93.95)	84.3–100.7	1 (3.7)	0 (0.0)	
Full blood count	Mean platelet volume (fL)	27	—	9.7 (9.05–10.4)	8.5–12.3	—	—	
Full blood count	Monocyte count (×10^9^/L)	28	0.1–0.6	0.38 (0.285–0.47)	0.05–0.9	1 (3.6)	1 (3.6)	
Full blood count	Neutrophil count (×10^9^/L)	27	1.8–6.3	4.01 (2.84–4.82)	1.08–6.34	1 (3.7)	2 (7.4)	
Full blood count	Nucleated red blood cells (×10^9^/L)	28	—	0 (0–0)	0–0.01	—	—	
Full blood count	Platelet count (×10^9^/L)	28	125–350	220 (189–308)	108–350	0 (0.0)	2 (7.1)	
Full blood count	Platelet distribution width (fL)	28	—	11 (9.525–12.85)	7.9–16.6	—	—	
Full blood count	Plateletcrit (%)	28	—	0.216 (0.18–0.295)	0.12–0.36	—	—	
Full blood count	RDW-CV (%)	28	11.5–14.5	12.7 (12.275–13.2)	11.7–15.4	1 (3.6)	0 (0.0)	
Full blood count	RDW-SD (fL)	28	—	42.8 (41.125–45.1)	38.2–57.1	—	—	
Full blood count	Red blood cell count (×10^12^/L)	28	M 4.3–5.8; F 3.8–5.1	4.36 (4.0425–4.7325)	2.79–5.57	0 (0.0)	5 (17.9)	
Full blood count	White blood cell count (×10^9^/L)	28	3.5–9.5	6.14 (5.2325–7.14)	1.35–9.28	0 (0.0)	2 (7.1)	
Humoral immunity and inflammation	Antistreptolysin O (IU/mL)	25	0–200	36.1 (25–48.4)	25–132	0 (0.0)	0 (0.0)	9
Humoral immunity and inflammation	C-reactive protein (mg/L)	25	0–5	2.85 (2.11–4.25)	1.52–192	6 (24.0)	0 (0.0)	
Humoral immunity and inflammation	Complement C3 (mg/dL)	25	79–152	96.2 (86.9–109)	66.2–159	1 (4.0)	1 (4.0)	
Humoral immunity and inflammation	Complement C4 (mg/dL)	25	16–38	24.4 (20.9–30.7)	11.5–55.7	3 (12.0)	1 (4.0)	
Humoral immunity and inflammation	High-sensitivity CRP (mg/L)	8	—	1.4 (0.6375–3.9425)	0.31–10.83	—	—	
Humoral immunity and inflammation	Immunoglobulin A (mg/dL)	25	82–453	232 (136–274)	37.5–446	0 (0.0)	3 (12.0)	
Humoral immunity and inflammation	Immunoglobulin E (IU/mL)	25	0–100	25.7 (11.5–44)	5–598	1 (4.0)	0 (0.0)	5
Humoral immunity and inflammation	Immunoglobulin G (mg/dL)	25	751–1560	1120 (915–1370)	520–1590	1 (4.0)	4 (16.0)	
Humoral immunity and inflammation	Immunoglobulin M (mg/dL)	25	46–304	64.4 (50.2–111)	10–271	0 (0.0)	4 (16.0)	
Humoral immunity and inflammation	Procalcitonin (ng/mL)	14	0–0.05	0.05 (0.05–0.05)	0.05–0.06	2 (14.3)	0 (0.0)	12
Humoral immunity and inflammation	Rheumatoid factor (IU/mL)	25	0–20	20 (20–20)	20–568	2 (8.0)	0 (0.0)	23
Biochemistry	Alanine aminotransferase (U/L)	28	M 9–50; F 7–40	13.1 (11.85–20.175)	6.5–51.8	2 (7.1)	1 (3.6)	
Biochemistry	Albumin (g/L)	28	40–55	40.2 (39–42.35)	34.2–48.9	0 (0.0)	12 (42.9)	
Biochemistry	Alkaline phosphatase (U/L)	28	M 45–125; F 35–100	78.5 (63.75–95.75)	43–197	5 (17.9)	1 (3.6)	
Biochemistry	Aspartate aminotransferase (U/L)	28	M 15–40; F 13–35	16.8 (14.9–21.375)	9.2–40.9	1 (3.6)	7 (25.0)	
Biochemistry	Cholinesterase (U/L)	28	—	7564 (6338.75–8352)	4883–13,938	—	—	
Biochemistry	Creatine kinase (U/L)	24	M 50–310; F 40–200	59 (41–97)	14–206	1 (4.2)	6 (25.0)	
Biochemistry	Creatinine (μmol/L)	28	M 57–97; F 41–73	62.5 (53.8025–71.25)	38.97–85.86	1 (3.6)	4 (14.3)	
Biochemistry	Direct bilirubin (μmol/L)	28	—	2.11 (1.0725–3.83)	0.44–14.96	—	—	
Biochemistry	Globulin (g/L)	28	20–40	25.1 (22.925–27.725)	16–32.9	0 (0.0)	5 (17.9)	
Biochemistry	Glucose (mmol/L)	25	3.9–6.1	5.56 (4.89–6.88)	3.22–16.15	10 (40.0)	2 (8.0)	
Biochemistry	Glycated haemoglobin (%)	7	4–6	6.7 (6.35–9.05)	5.9–10	6 (85.7)	0 (0.0)	
Biochemistry	HDL cholesterol (mmol/L)	12	≥1	1.09 (0.895–1.3375)	0.67–1.86	0 (0.0)	5 (41.7)	
Biochemistry	LDL cholesterol (mmol/L)	12	0–3.4	2.975 (2.6525–3.25)	0.91–4.17	3 (25.0)	0 (0.0)	
Biochemistry	Lactate dehydrogenase (U/L)	24	120–250	210 (161.5–226.5)	146–467	4 (16.7)	0 (0.0)	
Biochemistry	Total bile acids (μmol/L)	28	—	3.345 (1.9875–5.03)	0.58–8.75	—	—	
Biochemistry	Total bilirubin (μmol/L)	28	0–26	12.95 (8.65–15.33)	1.66–24.95	0 (0.0)	0 (0.0)	
Biochemistry	Total cholesterol (mmol/L)	13	0–5.2	4.93 (4.13–5.32)	2.64–12.36	5 (38.5)	0 (0.0)	
Biochemistry	Total protein (g/L)	27	65–85	65.6 (61.85–71.05)	53.5–76.3	0 (0.0)	12 (44.4)	
Biochemistry	Triglycerides (mmol/L)	13	0–1.7	1.7 (1.1–2.63)	0.94–12.98	6 (46.2)	0 (0.0)	
Biochemistry	Urea (mmol/L)	28	M 3.1–8; F 2.6–7.5	4.7 (4.1–5.525)	3.2–12.5	3 (10.7)	0 (0.0)	
Biochemistry	Uric acid (μmol/L)	28	M 208–428; F 155–357	298 (234.25–356.75)	160–474	5 (17.9)	0 (0.0)	
Biochemistry	VLDL cholesterol (mmol/L)	12	—	0.395 (0.225–1.16)	0.06–10.07	—	—	
Biochemistry	eGFR (mL/min/1.73 m^2^)	27	≥90	96 (87.045–106.64)	61.39–121.37	0 (0.0)	9 (33.3)	
Biochemistry	α-hydroxybutyrate dehydrogenase (U/L)	24	—	158.5 (124.5–170.75)	10–384	—	—	
Biochemistry	γ-glutamyl transferase (U/L)	28	M 10–60; F 7–45	23.4 (15.75–28.725)	12.5–120	4 (14.3)	0 (0.0)	
Biochemistry	Free fatty acids (mmol/L)	12	—	0.43 (0.315–0.535)	0.18–119	—	—	
Biochemistry	Mitochondrial aspartate aminotransferase, m-AST (U/L)	28	—	3.25 (2.3–4.875)	1.5–13.4	—	—	
Biochemistry	Creatine kinase-MB mass, CK-MB (ng/mL)	12	—	1.98 (1.3075–2.2075)	0.79–3.89	—	—	
Biochemistry	Non-HDL cholesterol (mmol/L)	9	—	3.79 (3.23–4.46)	1.19–11.69	—	—	
Coagulation	Activated partial thromboplastin time (s)	26	28–42	34 (31.45–37.275)	17.7–43.4	1 (3.8)	1 (3.8)	
Coagulation	D-dimer (mg/L FEU)	25	0–0.55	0.27 (0.22–0.36)	0.12–1.22	2 (8.0)	0 (0.0)	1
Coagulation	Fibrinogen (g/L)	26	2–4	3.605 (3–4.425)	2.5–6.86	8 (30.8)	0 (0.0)	
Coagulation	International normalised ratio	26	0.8–1.2	0.99 (0.9225–1.0275)	0.89–1.25	1 (3.8)	0 (0.0)	
Coagulation	Prothrombin time (s)	25	11–14.5	12.6 (12.2–13.1)	8.96–14.8	1 (4.0)	3 (12.0)	
Coagulation	Thrombin time (s)	26	14–21	17.5 (16.9–18.475)	15.4–21.4	1 (3.8)	0 (0.0)	

**Abbreviations:** CK-MB, creatine kinase-MB; eGFR, estimated glomerular filtration rate; HDL, high-density lipoprotein; LDL, low-density lipoprotein; MCHC, mean corpuscular hemoglobin concentration; Non-HDL, non-high-density lipoprotein; RDW-CV, red blood cell distribution width–coefficient of variation; RDW-SD, red blood cell distribution width–standard deviation; VLDL, very-low-density lipoprotein.

## Data Availability

De-identified data supporting these findings are available from the corresponding author on reasonable request, subject to institutional approval.
